# Creating *Mi*nimum *Ha*rm *P*ractice ( *MiHaP*): a concept for continuous improvement

**DOI:** 10.12688/f1000research.2-276.v1

**Published:** 2013-12-17

**Authors:** Ranjit Singh

**Affiliations:** 1 Department of Family Medicine, School of Medicine and Biomedical Sciences, University at Buffalo, NY, USA; 2 UB Patient Safety Research Center, University at Buffalo, NY, USA; 3 Department of Management Science and Systems, School of Management, University at Buffalo, NY, USA

## Abstract

The author asks for the attention of leaders and all other stakeholders to calls of the World Health Organization (WHO), the Institute of Medicine (IOM), and the UK National Health Service (NHS) to promote continuous learning to reduce harm to patients. This paper presents a concept for structured bottom-up methodology that enables and empowers all stakeholders to identify, prioritize, and address safety challenges. This methodology takes advantage of the memory of the experiences of all persons involved in providing care. It respects and responds to the uniqueness of each setting by empowering and motivating all team members to commit to harm reduction. It is based on previously published work on “Best Practices Research (BPR)” and on “Systematic Appraisal of Risk and Its Management for Error Reduction (SARAIMER)”. The latter approach, has been shown by the author (with *Agency for Healthcare Research and Quality (*AHRQ) support), to reduce adverse events and their severity through empowerment, ownership and work satisfaction. The author puts forward a strategy for leaders to implement, in response to national and international calls for Better health, Better care, and Better value (the 3B’s of healthcare) in the US *Patient Protection* and Affordable Care Act. ^^This is designed to enable and implement “ *A promise to learn- a commitment to act”.*  AHRQ has recently published “A Toolkit for Rapid-Cycle Patient Safety and Quality Improvement” that includes an *adapted* version of SARAIMER.

## Introduction

The September 2012 Institute of Medicine (IOM) report ^1^ called for higher quality at lower cost through leadership that fosters continuous learning. It makes ten important recommendations (e.g. “involve patients and families in decisions regarding health and health care, tailored to fit their preferences” and “continuously improve health care operations to reduce waste, streamline care delivery, and focus on activities that improve patient health”). The IOM very rightly asserts that there can be no quality without safety.

An August 2013 report ^2^ to the British Prime Minister by Britain’s National Advisory Group on the Safety of Patients in England, draws attention to the fact that the current state of patient safety is in “Crisis”. It calls for “ *A promise to learn- a commitment to act*” for improving the safety of patients in England. This report identifies fifteen problems including the use of “quantitative targets” without caution. Based on these, it makes ten recommendations (e.g. “The NHS should continually and forever reduce patient harm by embracing whole heartedly an ethic of learning” and “All leaders concerned with NHS healthcare-political, regulatory, governance, executive, clinical and advocacy – should place quality of care in general, and patient safety in particular, at the top of their priorities for investment, inquiry, improvement, regular reporting, encouragement and support”). Both reports make the vital statement that the power of bottom-up commitment for continuous learning for quality improvement is much greater than that of the top-down efforts that are based on rules, standards and enforcement. Bottom-up initiatives and acts will help fulfill the mission of the US Patient Protection and Affordable Care Act. We need to develop a facility that enables and helps implementation of “ *A promise to learn- a commitment to act*” ^2^. Providers are frustrated with the top-down management methods that are by their very nature mechanistic (Taylorism), as against humanistic (bottom-up) methods. Top-down methods include: external audits, practice profiles, quality and safety indicators, and trigger tools. These tools do not adequately recognize the problems involved with defining and quantifying harm ^3^.

Organizational changes required to reduce the huge global burden of harm in healthcare should take advantage of recommendations in these two reports, supported by previously published work by Mold and Gregory ^4^ on “Best Practice Research (BPR)” and Singh *et al.* on “Systematic Appraisal of Risk and Its Management for Error Reduction (SARAIMER)” ^5– 7^. The Agency for Healthcare Research and Quality ( *AHRQ*) has recently published “A Toolkit for Rapid-Cycle Patient Safety and Quality Improvement” that includes an adapted version of SARAIMER ^8^.

This paper presents a concept that embeds the BPR in SARAIMER for creating a sustainable and affordable methodology for continuously improving safety-based quality of care efficiently and effectively. The guiding principle in healthcare is that improvement science is not about developing a theory begging for application but rather is about understanding societal needs and wants, and engineering means of meeting them in an efficient manner that is sustainable in the never ending journey toward excellence.

## What is Best Practice Research (BPR) method in healthcare?

This is a bottom-up systematic study that identifies, narrates, combines and disseminates effective and efficient management strategies designed by and for practicing providers. The primary objective is to create a list of desirable (BEST) qualities followed by prioritizing them with stipulated minimum standards for each. This approach can help fill the gap (in knowledge of desirable qualities and of their prioritization) often left in medical school and residency training programs ^9^. This approach to research captures the collective experiences and wisdom of providers working in their unique settings. Development of BPR was motivated by frustration with the top-down management methods.

In general, the BPR method includes:

- Development of a conceptual model. This essentially captures all the contributing parts of the process, including an appropriate process flow diagram.
- Definition of “BEST” method. This creates a list of desirable qualities, prioritizing them and setting a minimum standard for each.
- Evaluation of the potential methods for each process.
- Combining best processes.
- Testing of the combined method.

Put forward by Mold and Gregory, this method invokes improvement science ^10^. Their goal is to find a solution for best practice. They provide examples in their 2003 paper ^4^. Their records showed positive responses from all participants in their research. Lessons learnt from their approach can build a strong house of quality on the triad of *Structure-Process-Outcome* put forward by Donabedian ^11^. This triad led the IOM in 2001 to form the list of six components of quality care namely: efficient, effective, timely, equitable, safe, and patient centered.

## What is Systematic Appraisal of Risk And Its Management for Error Reduction (SARAIMER)?

**(a): Introduction:** Despite numerous national and international calls for improvements in safety, healthcare progress has been painfully slow. In the US up to 200,000 avoidable deaths per year occur in primary and outpatient settings alone ^12, 13^. In the US alone nearly eleven million patients are harmed every year due to avoidable errors ^14, 15^. The associated costs are estimated to be $1 trillion/year out of a total healthcare annual budget of $3.2 trillion. Reduction of these is an ethical, societal, and fiscal imperative that demands priority from all stakeholders. Recently, various accreditation bodies have started to pay greater attention to the reduction of this huge burden of harm to patients. The US Accreditation Council of Graduate Medical Education (ACGME) has recently called for implementation of a “Next GME Accreditation System” ^16^. This current paper draws particular attention to “System-Based Practice” and “Practice-Based Learning and improvement”. These two competencies have, hitherto, received little attention despite their importance.

The most commonly used methods of measuring harm in healthcare, are based on error reports, external audits and profiles, quality and safety indicators, and trigger tools. The *Health Affairs* issue of April 2011 published a review showing that, for a specific setting and time, the total number of adverse events measured with voluntary reporting, AHRQ Safety Indicators and global trigger tool ^17^, were 4, 35 and 354 respectively in a total of 795 patient records.These different measures of the same reality obtained with different methods of observation testify to the fact that what we measure in healthcare is a function of how and why we measure. A statement that is often made in medical literature is: “if you cannot measure it you cannot improve it”. This is attractive and relevant in the engineering and manufacturing domains. In the science of healthcare, however, unconditional reliance on these so-called *measures* narrows the range and depth of our progress. Science of improvement ^10^ is a function of the complex Donabedian Triad of *Structure-Process-Outcome* ^11^. The prevalent reductionist approaches are a barrier to advances in outcome improvements such as patient harm reduction. It is relevant to recall the observation made by the Nobel Laureate Prigogine that “science is an expression of contemporary culture”.

These thought processes led the author and his team to develop SARAIMER, which captures the memory ^18, 19^ of providers and patients in each unique setting and empowers, enables, and motivates them at the point of care to make sustainable continuing improvements.

**Figure 1.**
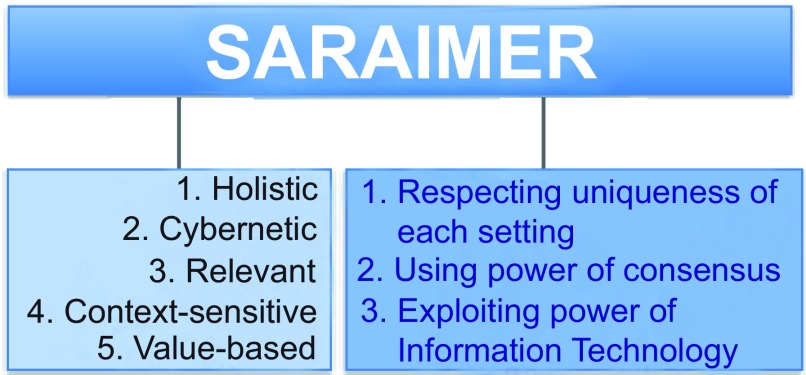
Supporters of Systematic Appraisal of Risk and Its Management for Error Reduction (SARAIMER).

**(b) Description of SARAIMER:** This systems approach to practice-based learning and improvement is built on three considerations and five principles as illustrated in Figure 1. SARAIMER, adapted from Failure Mode and Effects Analysis (FMEA), is designed to foster mutual respect, trust, understanding, collaboration, cooperation, and work and patient satisfaction. The approach has been demonstrated ^5, 6^, with AHRQ support, to reduce adverse events and their severity through empowerment and ownership.

**Figure 2.**
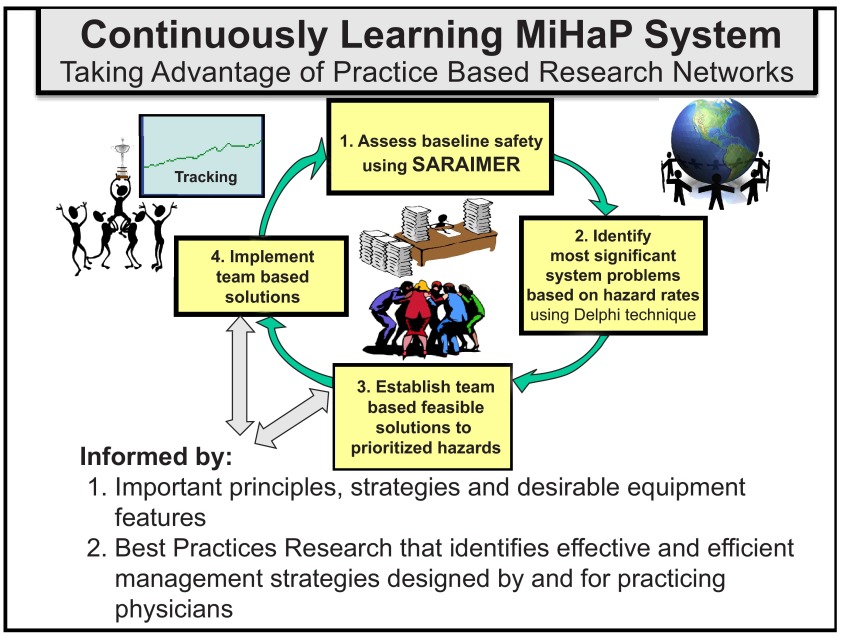
Cyclic SARAIMER-based harm reduction cycle.

This approach loops the science of observed systems with that of observing them resulting in a cyclic methodology as illustrated in Figure 2. In the concept proposed by the author the third stage in SARAIMER is enriched with BPR input. All the SARAIMER stages are briefly described ^5, 6^ below:

*Stage (1) Assess baseline safety with annonymous on-line survey*: The first step in this adapted FMEA process is to begin to understand the system of care in the setting. This is done by first identifying the various entities in the practice (such as the patient, provider, nurse, and chart), listing the main interactions between them, and then portraying them in a diagram. Memory of staff about hazards in each entity is captured with a specially designed instrument.

*Stage (2) Identify the most significant system problems using hazard analysis for identification of the most hazardous failure modes and prioritization*: The goal of the analysis is to rank the failure modes from the survey according to the size of their effects. This is followed by a web-based consensus forming process (Delphi Technique) the results of which are presented to staff in a graphic format. This results in team decisions regarding which hazards to address.

*Stage (3) Establish team based feasible solutions to prioritized hazards*: In light of the resources available and the capabilities of the unique setting, the teams develop solutions to address the prioritized vulnerabilities. These solutions are informed by: (a) established safety principles, (b) safety strategies and (c) desirable equipment safety design features. BPR-identified effective and efficient management strategies are invoked at this stage to enrich feasible solutions.

*Stage (4) Implementation of team based improvement interventions and tracking their effects*: The staff are helped to form implementation teams with clear allocation of responsibilities and time schedules. Along with this, the outcomes of the interventions are tracked with the aid of the software written for this purpose.

In his 2012 papers ^5, 6^ the author provides details of methodology of SARAIMER interventionin various offices. This intervention (without BPR enrichment) resulted in significant reductions in frequency and severity of harm to patients. Participants, including all staff, in *every* practice expressed great enthusiasm, and recommended wider dissemination of his team-based methodology because it encouraged and enabled them to continuously learn to reduce harm with greater work satisfaction.

**(c) Minimum Harm Practice (MiHaP)** (an acronym of *May I Have Your Attention Please!*) **by embedding BPR, and safety principles and strategies in SARAIMER**: At philosophical and improvement science levels, the development process for MiHaP must use a system engineering approach. Medical practices operate in fast changing, complex circumstances. They need to be able to adapt quickly, and to find order where there is disorder. Order helps to create continually learning practice.

**Figure 3.**
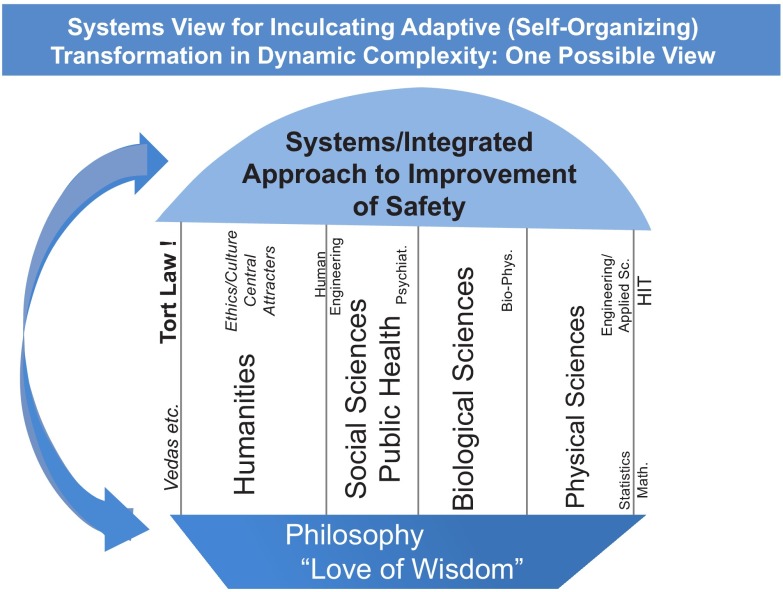
Systems thinking integrating information and knowledge from various domains to avoid reductionism.

It was in 1946 that T.S. Eliot asked: “Where is the wisdom we have lost in knowledge?, where is the knowledge we have lost in information?” Even today, we compartmentalize information and knowledge into different fields of science, such as physics, biology, mathematics, etc. In other words we tend to fragment our information and knowledge, thus retarding our progress to wisdom. The fragmentation in the US healthcare ‘non-system’ compounds the complexity. To bring about order we must avoid reductionism by adopting systems thinking so as to integrate information and knowledge, leading to wisdom that enables ethical advancements. This thought is expressed in Figure 3.

**Figure 4.**
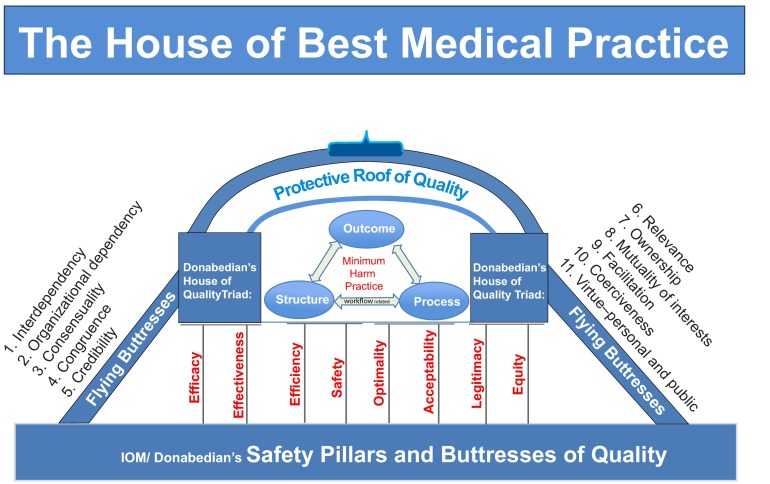
Donabedian house of quality on sound foundation and lateral supports.

**Figure 5.**
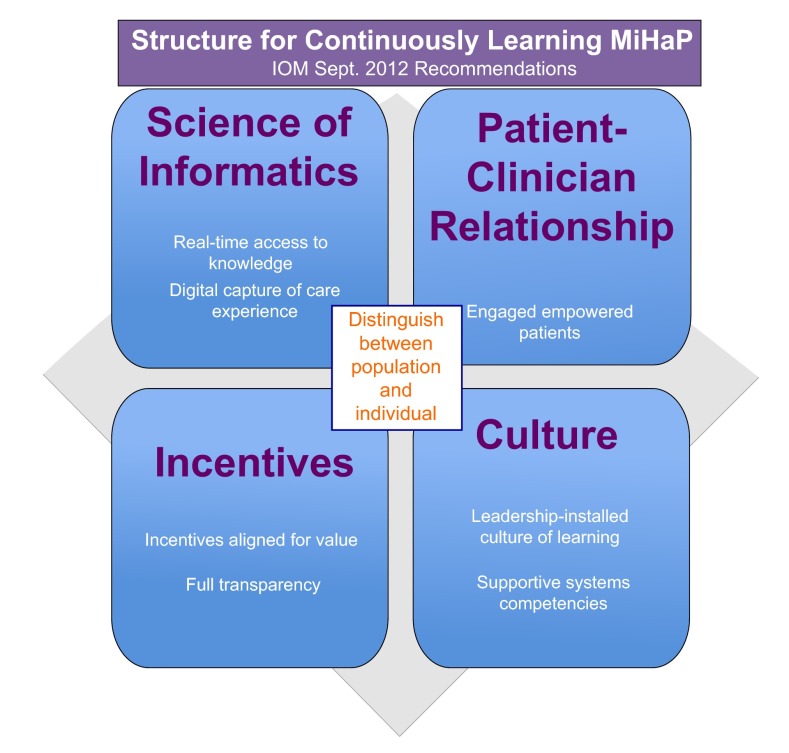
September 2012 IOM report recommended structure for continuous learning.

At a practical level, MiHaP should help form best medical practice built on Donabedian’s house of quality triad with attention to a sound foundation and lateral supports (flying buttresses) as shown in Figure 4. To make this house a continuously learning practice it should take advantage of the September 2012 IOM report recommendations: provide real-time access to knowledge by digitally capturing care experiences (e.g. BPR), engaging empowered patients, providing transparency with incentives aligned for value, and provide leadership that cultivates a culture of learning in system-based practice and practice-based learning and improvement. This is illustrated in Figure 5.

As described earlier, SARAIMER presents an operational framework in which can be embedded, as shown in lower part of Figure 2, not only the safety principles and strategies (including equipment features) but also effective and efficient best practice management strategies designed by and for providers and disseminated through Practice Based Research Networks (PBRNs). The overall objective of BPR has to be the delivery of safe, effective, efficient, equitable, timely and patient-centered care, as called for by IOM. These have to be supported by a caring organization that pays attention to continuity of care ^20^.

For harm reduction MiHaP has to be armed with sharp awareness of and attention to the following systemic threats of the process of care:

- Variability from patient to patient
- Inconsistency in the standards of care
- Poor interfacing (e.g. transition between settings)
- Lack of error-preventing barriers
- Lack of initiative to handle the unforeseen
- Use of inappropriate time constraints
- Use of a hierarchical culture in the system and
- Human fallibility – *to err is human*

**Figure 6.**
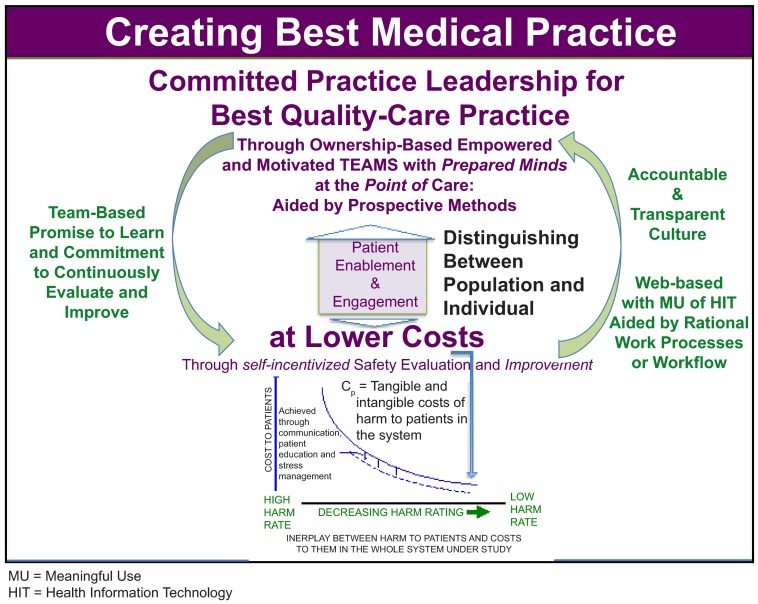
Concept For Minimum Harm Practice with its vital determinants.

As stated earlier, there are national and international calls for Better health, Better care, and Better value (3B’s of Healthcare). In response to the 2012 IOM report and the Britain’s National Advisory Group, the author of this paper proposes a concept for MiHaP portrayed in Figure 6. The details in this figure are a reflection of the complexity of care. SARAIMER is designed to inculcate order in this complexity by fostering mutual respect, trust, understanding, collaboration and cooperation as well as worker and patient satisfaction. The bottom part of this figure emphasizes the business case for improving safety, focusing on the costs of harm to patients. This figure captures the primary driving forces in best practice.

## Concluding remarks

A safe organization is a cost effective quality organization. A concept for a structured bottom-up methodology is put forward that motivates, engages, enables and empowers all stake holders to identify, prioritize, and address safety challenges. This methodology takes advantage of the memory of all team members involved in providing care. It respects and responds to the uniqueness of each setting by empowering and motivating all to a never ending commitment to harm reduction. A strategy is put forward for leaders to implement, in response to national and international calls for Better health, Better care, and Better value (3B’s of Healthcare). The author’s contention is that incorporation of BPR in SARAIMER will accelerate the journey to the desired outcome (reduced harm). This strategy is designed to enable and implement “ *A promise to learn- a commitment to act*” ^2^. It is useful to add that AHRQ has recently published “A Toolkit for Rapid-Cycle Patient Safety and Quality Improvement” that is based on an adapted paper version of SARAIMER.
