# Supplementary material for: Creating Minimum Harm Practice ( MiHaP): a concept for continuous improvement
Source: F1000Res. 2013 Dec 17;2:276. [Version 1] doi: 10.12688/f1000research.2-276.v1 (PMC3968896; doi:10.12688/f1000research.2-276.v1)
Supplement: Peer review history

# Peer review: Referee response for version 1

**Published:** 2014-02-05

**Authors:** Christian Gericke

**Affiliations:** 1 Wesley Research Institute and University of Queensland, Brisbane, Australia

This is a well written conceptual paper, proposing a bottom-up methodology for quality and safety projects in healthcare that has real potential for widespread use throughout healthcare systems.

I have read this submission. I believe that I have an appropriate level of expertise to confirm that it is of an acceptable scientific standard.

# Peer review: Referee response for version 1

**Published:** 2014-01-21

**Authors:** John Hickner

**Affiliations:** 1 Family Medicine, University of Illinois Hospital and Health Sciences System, Chicago, IL, USA

I am a U.S. family physician, and I have been aware of Dr. Singh's important work in safety and quality for nearly a decade. I am also aware of the "Best Practices" model for improvement championed by Mold and others. Both of these approaches are pragmatic and build on the knowledge of those at the front line, much like the Toyota model has done for industry. This manuscript is a well written summary of these two improvement methods, firmly grounded in the context of calls for healthcare improvement in the U.S. and the U.K.

There is no question that improvement in healthcare will come from both top down and bottom up approaches. So far, the balance of improvement efforts have been top down. To the extent that practitioners and their support staff can become engaged in improvement efforts, the pace will accelerate. Organizations that are able to harness the knowledge and spirit of their employees are likely to forge ahead of the rest in quality and safety. We are already seeing this in the U.S.

I have read this submission. I believe that I have an appropriate level of expertise to confirm that it is of an acceptable scientific standard.
